# Single-walled carbon nanotubes as a nano-weapons against biofilm of *Pseudomonas aeruginosa*

**DOI:** 10.3389/fmicb.2026.1791060

**Published:** 2026-04-10

**Authors:** Majed Ahmed Al-Shaeri, Mohammad Oves

**Affiliations:** 1 Department of Biological Sciences, Faculty of Science, King Abdulaziz University Jeddah, Saudi Arabia; 2 Environmental Protection and Sustainability (EPS) Research Group, Faculty of Science, King Abdulaziz University, Jeddah, Saudi Arabia; 3 Center of Excellence in Environmental studies, King Abdulaziz University Jeddah, Saudi Arabia

**Keywords:** antimicrobial activity, biofilm, MBC, MIC, *P. aeruginosa*, single-walled carbon nanotubes, SWCNTs

## Abstract

**Background/introduction:**

The emergence of antimicrobial resistance in bacterial biofilms represents a growing global healthcare burden, necessitating the development of novel agents with alternative mechanisms of action.

**Methods:**

In the present study, we evaluated the antibacterial and antibiofilm potential of single-walled carbon nanotubes (SWCNTs) against *Pseudomonas aeruginosa*, a clinically significant opportunistic pathogen notorious for its robust biofilm-forming capacity and intrinsic resistance profile. Antimicrobial activity was assessed using disc diffusion and broth microdilution assays, while biofilm inhibition was quantified by crystal violet microplate assays. Scanning electron microscopy (SEM) and Fourier-transform infrared spectroscopy (FTIR) analyses were performed to elucidate the underlying antibacterial mechanism.

**Results:**

SWCNTs exhibited potent concentration-dependent bacteriostatic and bactericidal effects, with a minimum inhibitory concentration (MIC) and minimum bactericidal concentration (MBC) of 62.5 and 125 μg/mL, respectively. Zones of inhibition ranged from 14.5 ± 0.30 mm to 22.0 ± 0.57 mm across concentrations of 4–16 mg/mL (*p* ≤ 0.05). In biofilm inhibition assays, planktonic growth (OD_470_) was markedly reduced from ≈0.32 ± 0.01 in untreated controls to ≈0.05 ± 0.01 at 200 μg/mL, corresponding to a maximum biofilm inhibition rate of 85.5%. SEM imaging revealed pronounced morphological disruption of *P. aeruginosa* cell walls, including membrane deformation, surface roughening, and loss of cellular integrity upon SWCNT treatment, indicative of direct physical interaction between the nanotubes and bacterial membranes. FTIR analysis further corroborated these findings, demonstrating characteristic spectral shifts in functional groups associated with bacterial membrane lipids, proteins, and polysaccharides.

**Discussion/conclusion:**

These spectral changes suggest physicochemical interactions that compromised membrane stability and disrupted biofilm matrix integrity. Collectively, these findings support a proposed mechanism whereby SWCNTs exert their antibacterial effect through direct membrane perturbation, interference with biofilm extracellular polymeric substances (EPS), and inhibition of early-stage biofilm adhesion and maturation.

## 1 Introduction

Antimicrobial resistance (AMR) has emerged as one of the most pressing global public health crises of the 21st century. AMR threatening the efficacy of conventional antibiotic therapies and contributing significantly to morbidity and mortality worldwide. A central driver of this resistance is the capacity of the pathogenic bacteria to form biofilms. Biofilm is a structured, surface-adhered microbial communities encased within a self-produced extracellular polymeric substances (EPS) matrix (Flemming and Wingender, 2010). The bacteria covered by layer of EPS confer dramatically enhanced tolerance to antimicrobial agents compared to their planktonic counterparts (Abdalla et al., 2021). Biofilms act as a formidable diffusion barrier that impede antibiotic penetration, facilitate horizontal gene transfer of resistance determinants, and provide shield to bacteria for host immune responses, making biofilm-associated infection significantly difficult to eradicate with current therapeutic regimens (Azeem et al., 2025; Ugwu et al., 2025). The inadequate pipeline of novel antibiotics, combined with the accelerating evolution of multidrug-resistance (MDR) strains, underscores the urgent need for alternative antimicrobial strategies with distinct mechanisms of action (Elshobary et al., 2025).

*Pseudomonas aeruginosa* is among the most clinically significant biofim-forming pathogens globally and has been designated a WHO Priority 1 Critical pathogen, for which the development of new therapeutic strategies is considered necessary (World Health Organization, 2022). It is clinical prominence stems from its intrinsic resistance to multiple antibiotics, remarkable genomic plasticity, and exceptional capacity to from structurally complex biofilms. The biofilm EPS matrix of *P. aeruginosa* is biochemically distinct from that of commonly studied organisms such as *E. coli, S. aureus*, and *B. subtilis*, being characterized by a thick alginate matrix. The *P. aeruginosa* extracellular DNA (eDNA) scaffolding, and pel/psl polysaccharide architecture that together create an exceptionally robust diffusion barrier (Gong et al., 2024). These structural features render *P. aeruginosa* biofilms particularly refractory to conventional antimicrobial agents and necessitate the investigation of novel nanomaterial-based approaches capable of disrupting biofilm integrity at the molecular level.

Carbon-based nanomaterials, and Single-Walled Carbon Nanotubes (SWCNTs) in particular, have attracted considerable scientific interest as a promising next-generation antimicrobial agents owing to their unique combination of physiochemical properties including high aspect ratio, nanoscale diameter, surface activity, and the capacity for direct physical interaction with bacterial membrane (Alfei and Schito, 2025). In initial landmark investigation by Kang et al. (2007) demonstrated potent bactericidal activity of SWCNTs against planktonic *E. coli* through direct membrane puncture and physical disruption of cellular integrity. Further studies by the same group and others established that oxidative stress also contributes to SWCNT-mediated cytotoxicity, as evidenced by the upregulation of bacterial oxidative stress response genes, these associated gene *soxRS* and *oxyR* regulons, upon exposure to both SWCNTs and MWCNTS (Kang et al., 2008). Furthermore, studies have demonstrated additional antibacterial strategies, including coating of SWCNTs with polyvinylpyrrolidone iodine (PVPI) to enhance surface antimicrobial activity, and incorporation of MWCNTs into polymer or metal ion composite materials to inhibit bacterial attachment and biofouling (Jing et al., 2018; Rabie et al., 2022). Overall, these studies have established a mechanistic framework encompassing direct membrane damage, lipid peroxidation, and reactive oxygen species (ROS)-mediated oxidative injury as the principal pathways through which CNTs exert antimicrobial effects against planktonic bacteria.

However, despite this body of evidence, critical knowledge gaps remain. The vast majority of prior mechanistic studies have been conducted using planktonic bacterial models and do not address the fundamentally distinct challenge posed by mature, three dimensional. EPS-encased biofilm communities (Abebe, 2020; Mazza, 2016). The penetration of SWCNTs through the dense EPS matrix of *P. aeruginosa* biofilms, their interaction with biofilm structural components including polysaccharides, structural proteins, and membrane lipids, and their capacity to disrupt biofilm architecture and inhibit biofilm formation remain poorly characterized. Furthermore, no previous study has employed Fourier-transform infrared (FTIR) spectroscopy to characterize the molecular-level interactions between SWCNTs and *P. aeruginosa* biofilm matrix components, leaving the physicochemical basis of any anti-biofilm activity unresolved. Scanning electron microscopy (SEM)-based ultrastructural analysis of SWCNT-treated *P. aeruginosa* biofilms, capable of revealing morphological disruption at the cellular and matrix levels, has similarly not been reported in a dedicated and systematic manner for this clinically critical species.

The present study therefore addresses three clearly defined research gaps: (i) the species-specific anti-biofilm activity of SWCNTs against *P. aeruginosa* as a dedicated and systematic investigation, distinct from prior generalized planktonic antimicrobial studies; (ii) the mechanistic characterization of SWCNT interaction with the *P. aeruginosa* EPS matrix and cellular architecture using complementary SEM ultrastructural analysis and FTIR spectroscopic evidence of molecular-level SWCNT-biofilm interactions; and (iii) a quantitative assessment of SWCNT-mediated biofilm inhibition using validated microplate-based assays. Antibacterial properties were further characterized by agar disc diffusion and broth microdilution assays to determine minimum inhibitory concentration (MIC) and minimum bactericidal concentration (MBC), alongside evaluation of concentration- and time-dependent inhibition kinetics. Together, these contributions establish a novel mechanistic and experimental framework for understanding SWCNTs as targeted nano-enabled agents against *P. aeruginosa* biofilm, rather than as generic broad-spectrum antimicrobials, and provide a rational basis for their further development in biofilm-associated infection control and nanomedicine applications.

## 2 Materials and methods

### 2.1 Materials and reagents

An SWCNT stock (1 mg L1; Sigma-Aldrich; catalog 704121; manufacturer’s specifications, diameter 1.1 nm length 0.5– 100 mm; G/D Ratio: At ≥ 15 (Raman 633 nm), its carbon basis was ≥ 90% (≥ 77% as carbon nanotubes) and was prepared in distilled water using 0.02% Suwannee River natural organic matter. An ultrasonic bath was used to disperse the SWCNT stock for 2 h before use.

### 2.2 Preparation and characterization of SWCNTs

A stock solution of single-walled carbon nanotubes (SWCNTs) was prepared by dispersing 1 mg L^–1^ of SWCNTs with a diameter of 1.1 nm and lengths ranging from 0.5 to 100 μm in distilled water. To facilitate dispersion, Suwannee River Natural Organic Matter (0.02% SRNOM) was used as a dispersant. Prior to use, the SWCNT stock was sonicated in an ultrasonic bath for a duration of 2 h. Stock suspensions were then examined using scanning and transmission electron microscopes to assess the characteristics of the SWCNTs. Further characterization and measurement of the SWCNTs under exposure conditions were conducted using dynamic light scattering and zeta potential analysis with a Malvern Nano-ZS Zetasizer.

### 2.3 Microbial strains and media

The Gram-negative pathogen *P. aeruginosa* (ATCC 27853) bioreporter bacterial strain was obtained from the Environmental Protection & Sustainability Laboratory (EPS), King Abdulaziz University. The strain was revived from the glycerol stock and cultured in Nutrient-Broth (NB) medium. The primary culture was subsequently incubated with agitation at 150 g and a temperature of 30°C for 24 h to promote propagation. Additionally, the antimicrobial activity of CNTs against *P. aeruginosa* was tested on Muller Hinton media at various concentrations.

### 2.4 Disk diffusion method

The bacterial strains were inoculated on the Mueller Hinton Agar Media (MHA) at a concentration of 10^7^ colony-forming units/mL. Three different concentrations of SWCNTs (4, 8, and 16 mg/mL) were impregnated into the SWCNTs, and a 7 mm paper filter disc was placed onto the agar impregnated with the SWCNTs. The SWCNTs were left to diffuse into the medium for 30 min at room temperature. SDW was the negative control. After 24 h at 37°C, these plates were incubated. The zone of inhibition was determined by the mean of trials undertaken in triplicate and the SD of the same. It is acknowledged that agar disk/well diffusion assays were originally developed and validated for water-soluble antibiotics and their direct application to carbon nanotube suspensions carries inherent methodological limitations. SWCNTs, due to their hydrophobic sp^2^ carbon surface, tendency to form colloidal aggregates rather than true molecular solutions, and physical dimensions approaching or exceeding the agar pore size (∼100–200 nm), do not diffuse freely through solid agar media in the manner assumed by the Kirby-Bauer interpretive framework (Bahr and Tour, 2002; Pluen et al., 1999). Consequently, zones of inhibition observed in the present study are more accurately interpreted as reflecting direct surface-contact antimicrobial activity at the disk–agar interface rather than as diffusion-gradient-mediated inhibitory concentration equivalents. These data should therefore be considered as qualitative and semi-quantitative screening evidence only, and potency determinations are based on the quantitative broth microdilution MIC and biofilm inhibition (MBIC) assay data reported (Balouiri et al., 2016; Hajipour et al., 2012).

### 2.5 Estimation of minimum inhibition concentration

An additional test was undertaken to determine the minimum inhibitory concentration for the samples of SWCNTs that exhibited antimicrobial activity during the antibacterial screening. MICs are often determined on 96 well microtiter plates in micro broth dilution tests. MICs were done using the micro-diluted Broth Technique as outlined by NCCLS to determine the MICs of the chemical on the examined bacteria. The incubation was carried out at 24 h at 37°C. Five microliter of Resazurin sodium solution (R7017 Sigma-Aldrich) was added to each well, after incubation. Column 11, which has only the media, tells us we had no plates contaminated during the wash setup. In column 12, there is a positive control containing exclusively cultivated bacterial stains. Columns 1 through 10 represent serial dilution of SWCNTs in media from 1 to 0.002 mg/mL. This method provides MICs with a high degree of precision and overcomes the large color and solubility problems that can thwart growth determinations with several substances (Agarwal et al., 2021). Furthermore, MICs determined in separate in triplicate flask with selected dose amount of nanomaterials and including adjustment of cultures to a standardized turbidity (0.5 McFarland standard) and subsequent dilution to achieve a final inoculum of approximately (e.g., 5 × 105 CFU/mL) in each cultured flask, following CLSI guidelines (Humphries et al., 2018).

### 2.6 Estimation of minimal bactericidal concentrations

The lowest concentration of samples treated with SWCNTs at which inoculated bacteria are killed are defined as the minimal bactericidal concentrations. The MIC microplate contents showing no bacterial growth were spread on nutrient agar plates with 10 μl of medium and re-inoculated into an incubator for 24 h at 37°C. A well was considered negative for growth and reported as the MBC when colony counts were < 5 (Elshikh et al., 2016).

### 2.7 Biofilm inhibition assay

**FIGURE 1:**
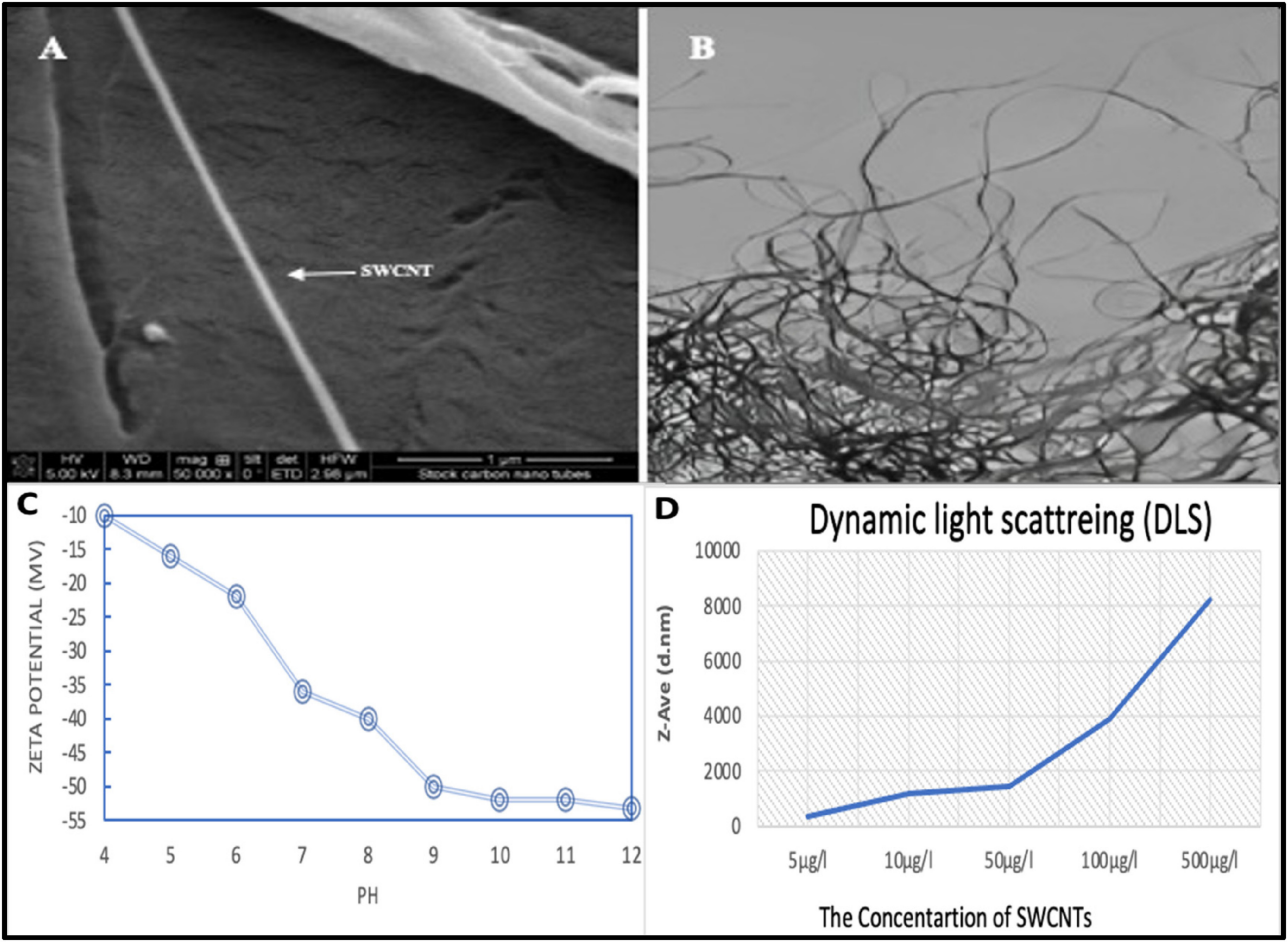
**(A)** An illustration from a scanning electron microscope (SEM) of single-walled carbon nanotubes (SWCNTs) that shows the shape of each nanotube on the substrate. **(B)** A transmission electron microscopy (TEM) picture that shows tangled networks of SWCNTs. **(C)** The zeta potential of SWCNTs changes with pH, showing that the surface charge becomes more negative as the pH rises. **(D)** Dynamic light scattering (DLS) analysis revealing the hydrodynamic diameter (Z-Ave) of SWCNTs in suspension at different concentrations.

The SWCNT’s effectiveness in inhibiting biofilm formation was tested using a microplate reader assay. Firstly, the biofilm formation by *P. aeruginosa* was confirmed in Figure 1. After incubation, the bacteria were cultured overnight and added to 150 μL of fresh NB broth. To this, 50 μL of SWCNTs were added following concentrations 50, 100, 150, and 200 μg/mL, and placed at 37°C for 24 h in the incubator. Following incubation, microplates were washed with phosphate-buffered saline at pH 7.4 to remove any cells that may be present and may influence the experiment results. 200 μL of 0.1% crystal violet solution was placed in the wells for 15–20 min to stain the biofilm layer. Next, it drained the crystal violet solution and applied 200 μL of 95% ethanol to the wells in Figure 3. The biofilm formation was then calculated using a plate reader by measuring the 570 nm absorbance by using Agilent BioTek Synergy HTX Multi-Mode Reader (Jevons and Quain, 2021).

### 2.8 Bacterial biofilm fixation and SEM investigation

This protocol enables accurate, high-quality observations and imaging of *Pseudomonas* biofilms under SEM. For scanning electron microscopy of *Pseudomonas* biofilms, type specimens were regulated on sterile coverslips or similar surfaces maintained in the appropriate culture medium to support biofilm formation. Generally, after 24–48 h of incubation, the coverslips were removed very delicately and washed with PBS to eliminate unattached cells. The fixation of biofilm was done using 2.5% glutaraldehyde solution prepared in phosphate-buffered saline PBS for 2–4 hours at 4°C to maintain cell structures. After fixation, the samples were washed three times with PBS to eliminate additional fixatives in the samples. Further, a dehydration series was performed with samples by placing them into 30, 50, 70, 90, and 100% ethanol solutions for 10–15 min for each concentration to extract water from the samples. Once dehydrated, the samples were dried using a critical point drying technique to prevent the destruction of the microstructures, followed by mounting them on aluminum stubs using carbon adhesive tape. Samples in mounted biofilms were prepared for SEM analysis by applying a thin layer of gold or platinum coating with the sputter coater for conduction. Last, the samples were viewed under scanning electron microscopy at the appropriate magnifications to analyze the biofilm organization, bacterial shapes, and additional polymicrobial biofilm matrixes.

Cultivate *Pseudomonas* sp. bacterial culture to the specified turbidity and formulate a suspension of single-walled carbon nanotubes (SWCNTs). Introduce the SWCNTs to the bacterial culture and incubate to facilitate contact and biofilm development. Segment the samples into two categories: one untreated and the other subjected to 5 mM EDTA, which serves as a positive control to prevent or disperse the biofilm matrix. Incubate both groups overnight to promote biofilm formation and interactions among SWCNTs, bacteria, and EDTA. Isolate bacterial cells and biofilms using centrifugation or direct sampling, subsequently employing a suitable fixative, such as glutaraldehyde, to maintain the integrity of the cell and biofilm structures. Dehydrate the materials using a graded ethanol series, followed by critical point drying or air drying. Affix the desiccated samples onto SEM stubs and apply a conductive coating, such as gold or platinum, using sputter deposition. Examine the samples with scanning electron microscopy, acquiring pictures at different magnifications. Images lacking EDTA treatment should exhibit SWCNT adhesion and breakdown of the bacterial membrane, whereas images with 5 mM EDTA treatment should demonstrate biofilm disruption and less bacterial aggregation. This procedure facilitates a comprehensive analysis of SWCNT and bacterial interactions, as well as the impact of EDTA on biofilm stability.

### 2.9 FTIR analysis of material functional group interaction with bacteria

A suspension of single-walled carbon nanotubes (SWCNTs) was formulated at a concentration of 100 micrograms per milliliter in a suitable sterile buffer or medium. To this suspension, 0.1 milliliters of *Pseudomonas* sp. bacterial culture, calibrated for turbidity to a specified optical density, was incorporated. The combination was incubated overnight at 37°C to facilitate contact between the SWCNTs and the bacteria. Following incubation, the mixture was centrifuged at 5,000 rpm for 10 min to sediment the biomass and associated nanotubes. The supernatant was meticulously discarded, and the pellet was desiccated in a vacuum oven at 70°C until thoroughly dry, usually for many hours or overnight. The dried pellet was subsequently pulverized with spectroscopic grade potassium bromide (KBr) powder to produce a fine, uniform mixture appropriate for FTIR measurement. A mixture of approximately 1–2 mg of the sample and 100 mg of KBr was compressed into a thin pellet. The pellet was positioned in the FTIR spectrometer sample holder, and the FTIR spectrum was obtained throughout the region of 4,000–400 cm^–1^. The resultant spectrum was examined to discern characteristic peaks associated with the SWCNT structure and potential interactions with the bacterial biofilm.

### 2.10 Statistical analysis

All antibacterial and anti-biofilm experiments were performed in triplicate across a minimum of three independent biological replicates (*n* = 3), conducted on separate occasions using freshly prepared bacterial cultures and material suspensions. Results are expressed as mean ± standard deviation (SD), as reflected in the error bars presented in all figures. The zone of inhibition diameters (Figure 1A) were measured independently for each replicate, and mean values with SD are reported.

Statistical comparisons between experimental groups were performed using one-way analysis of variance (ANOVA) followed by Tukey’s *post-hoc* test for multiple comparisons, using (OriginPro). A *p*-value of < 0.05 was considered statistically significant. Where applicable, significant differences between groups are indicated in the figures. The reproducibility of the results was confirmed across all independent experimental runs, with consistent trends observed in antibacterial activity across replicates, supporting the reliability of the reported findings.

## 3 Results

### 3.1 Characterization of SWCNTs

The SEM micrograph (Figure 1A) shows individual long filamentous structures with smooth surfaces confirming the successful synthesis and deposition of discrete SWCNTs on the substrate without any significant impurities or debris. As depicted in the TEM image (Figure 1B), a dense entanglement of bundles of nanotubes indicates the formation of a three-dimensional (3D) network of single-walled carbon nanotubes (SWCNTs) with a high aspect ratio and good innertubes contacts. Importantly, this is advantageous for surface-driven interactions that occur in aqueous media.

The zeta potential profile (Figure 1C) exhibits a monotonic decrease from approximately −10 to 10 mV at pH 4 to around −55 to 55 mV at pH 12. This shows that the negative surface charge progressively increases with alkalinity and that there is no isoelectric point within the studied pH range. This trend indicates that there is good electrostatic stability at neutral to alkaline pH, and the lower magnitude at acidic pH suggested a higher tendency to aggregate at low pH.

The hydrodynamic diameter (Z Ave) of SWCNTs, as estimated using dynamic light scattering analysis, increases sharply from a low-value micrometer range at concentrations of 5–10 μg/L to several micrometers at 500 μg/L (Figure 1D). The steep increase in Z Ave at high loadings indicates large concentration of SWCNTs and formation of large SWCNT clusters due to greater particle–particle collision frequency and inter-tube interaction.

### 3.2 Antibacterial test using agar diffusion assay

**FIGURE 2:**
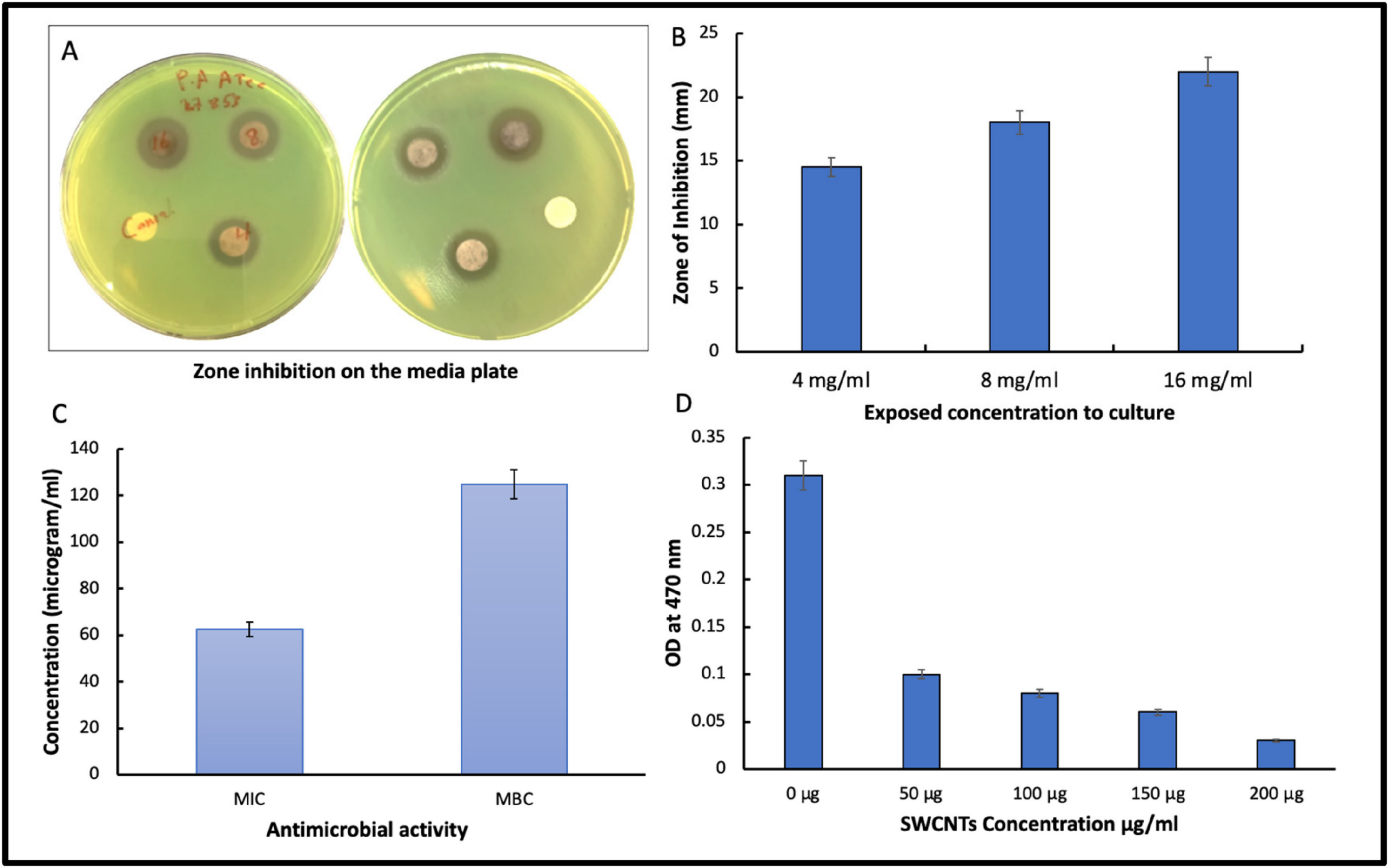
**(A)** Zone of inhibition (mm) of SWCNTs at 4, 8, and 16 mg/mL by agar disk diffusion assay against *P. aeruginosa* on media plate **(B)** Zone of inhibition (mm) of SWCNTs at 4, 8, and 16 mg/mL presentation by bar diagram. **(C)** The minimum bactericidal concentration of SWCNTs and the minimum inhibitory concentration against *P. aeruginosa*. **(D)** Determination of Anti-biofilm activity of SWCNTs at different optical density of *P. aeruginosa*.

Figure 2 suggests that SWCNTs show a clear, dose-dependent antibacterial activity, as evidenced by the well-defined inhibition zones and low MIC/MBC values, which can significantly inhibit bacterial growth at a high concentration. As illustrated in Figure 2A, the agar diffusion plates display a crucial observation of distinct zones of inhibition surrounding the wells containing the SWCNTs. Such a characteristic feature indicates that the SWCNTs are effective in restraining growth of the test bacterium. This effect is very much higher when compared to other areas of the same plate which were left untreated. The clear-cut circular zones imply the occurrence of a diffusible antimicrobial effect, indicating that contact mediated or released factor toxicity acts on planktonic phase and early biofilm cells.

An analysis showing the numeric (Figure 2B), with the diameter of the inhibition zone increased from 15 mm about at 4 mg/mL to 22 mm about at 16 mg/mL. Thus, it gives a strong enhanced concentration dependent anti-bacterial activity. The replicates vary very little, indicating reproducibility of the response at all doses tested.

The MIC and MBC values in panel C show that bacterial growth can be visibly stopped with a relatively low SWCNT concentration but its complete bactericidal activity requires around double that concentration. This MIC-MBC separation indicates a bactericidal action at higher concentrations, and we suggest that the action may switch from inhibition of growth to killing as the nanotube burden increases.

The decrease in optical density at 470 nm becomes abrupt as the concentration of SWCNT is increased from 0 to 200 μg/mL. This means a substantial drop in viable cell density. When looking at the higher concentration usage, the OD values are reaching their baseline levels.

### 3.3 Biofilm inhibition assay

**FIGURE 3:**
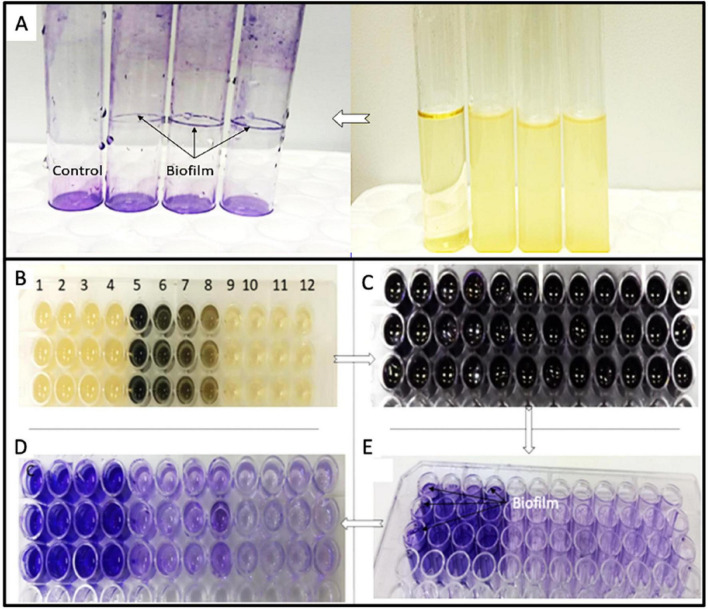
Results of test for biofilm formation by *P. aeruginosa.* Steps of biofilm inhibition assay. **(A)** Determination of Anti-biofilm activity of SWCNTs against *P. aeruginosa*. Each row **(B–E)** represents a triplicate of bacteria. Columns 1–4 represent the control cultured strain only, while columns 5–8 contain different concentrations of the SWCNTs sample (200, 150, 100, and 100 μg/mL) mixed with media. Columns 9–12 indicate the presence of media to confirm the absence of contamination on the plate.

The image demonstrates strong biofilm formation by the test organism in tubes and microtiter wells, while the crystal violet assay distinguishes wells that show biofilm formation from those that are planktonic/negative controls. Figure 3A displays the visible formation of biofilm in glass tubes. Upon decanting the planktonic culture, a heavy crystal violet stain appears as a dense ring and film on the inner walls of inoculated tubes. The uninoculated control tube exhibits no sign of a ring or film, only a uniform light stain of the medium with no adherent layer. The bright color at the air-liquid interface and on the tube wall indicates that stable, surface-attached biofilm has developed here, unlike sedimented cells which would be found uniformly spread on the tube bottom. Figures 3B–E shows demonstration of biofilm detection in microtiter 96 well plates by the microtiter crystal violet method In panel B, the middle columns of wells become dark after incubation to reflect the accumulation of high biomass, whilst the pale wells along the edge are uninoculated or corresponding to weakly adherent controls. High biomass wells and poor ones vary distinctly in their colors. The wells which are strongly positive after washing (Figure 3D) with crystal violet staining (Figure 3C) show dense purple staining and in panel E reproducible biofilm formation is observed where wells show a contiguous block of positive.

As seen in Figure 3A, the SEM–EDS analysis presents an irregular and densely coated surface. The subject of the map is mainly carbonaceous material that masks the cells. In Figure 3B, the corresponding EDS spectrum displays only the peaks for carbon and oxygen visible. Elemental analyses of the layer on the cells show 78 wt% (82 @%) carbon and 22 wt% (18 @%) oxygen. This further confirms the presence of a carbon rich SWCNT layer in intimate contact with the biological substrate. As shown in panel C, the cells treated with 50, 100, and 200 μg/mL SWCNTs were subjected under 15K×, 30K×, and 60K× (SEM). The cell surfaces were examined under a phase contrast microscope to observe any signs of corrugation or roughing up. At 50 μg/mL, with roughening and partial wrinkling of the cell surface, an early organization of membrane corrugation is revealed. As for the samples exposed to 100 μg/mL, there was more collateral and fragmented architecture, which is consistent with the devastating envelope disruption. Also, the samples treated at 200 μg/mL completely lost their native morphology, and so on.

**FIGURE 4:**
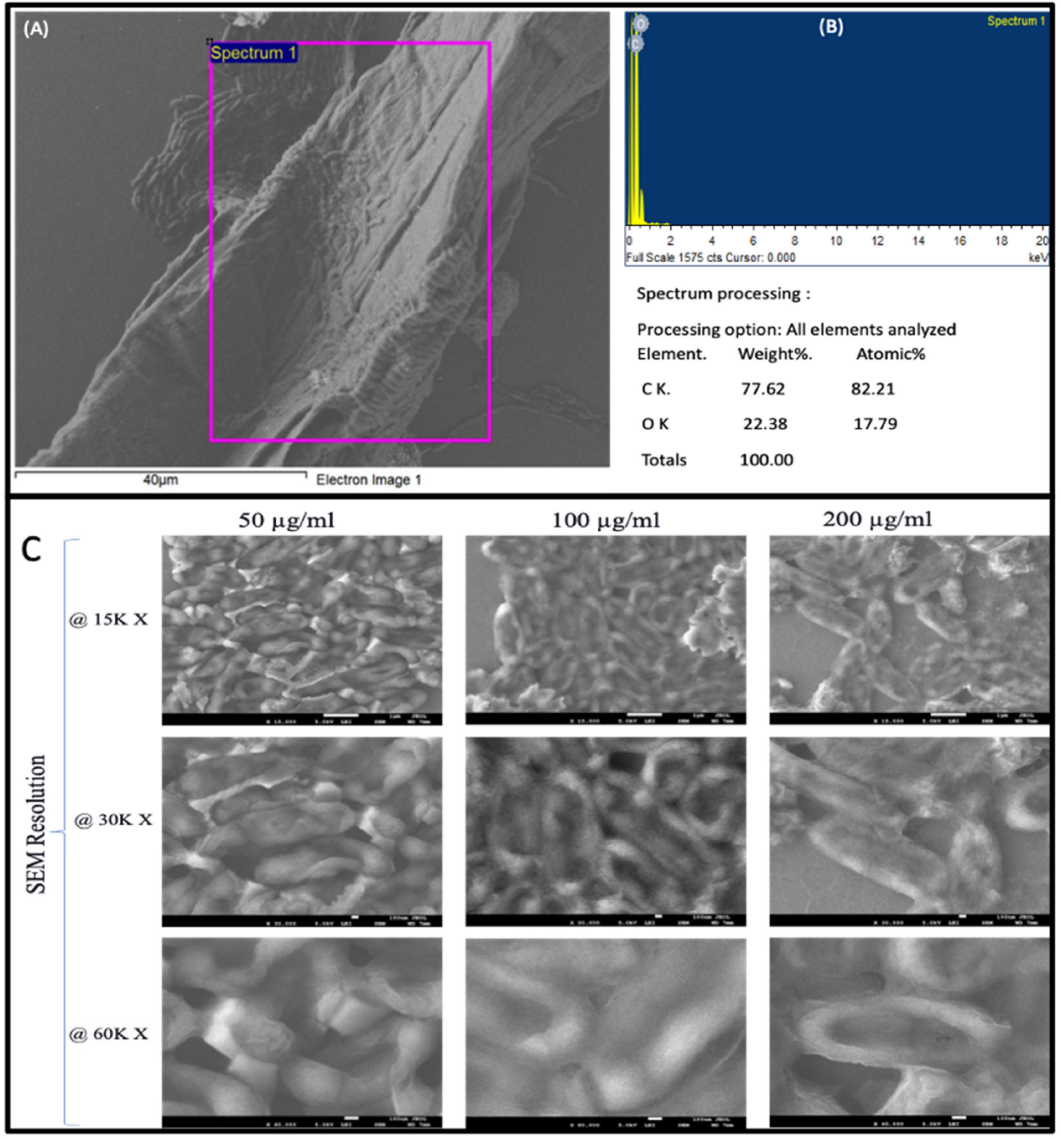
SEM image **(A)** showing the attachment of single-walled carbon nanotubes (SWCNTs) to bacterial cells. The purple box indicates the area analyzed for elemental composition using energy-dispersive X-ray spectroscopy (EDS). Spectrum 1 **(B)** reveals the elemental distribution within the selected area, highlighting the predominant presence of carbon and oxygen. The elemental analysis table confirms the weight and atomic percentages of the detected elements, demonstrating the carbon-rich nature of the nanotubes associated with the bacterial surface. **(C)** Biofilm of *P. aeruginosa* in presence of different concentration of SWCNT, 50, 100, and 200 μg/mL at different resolution 15, 30, and 60 K of bacterial cell distortion observation view.

The effect of single-wall carbon nanotubes (SWCNTs) on the biofilm of *Pseudomonas aeruginosa* reveals that SWCNTs can penetrate bacterial cells and alter the bacteria biofilm physically and chemically in a dose-dependent manner. The bacterial cultures were grown in the absence of SWCNTs but exposed to 50 μg of SWCNTs in culture, resulting in partial disruption of the biofilm of noticeable but limited damage to the extracellular matrix and bacterial cells. At a concentration of 100 μg, the effects were further magnified; in addition to morphological changes, the cell damage was also evident, showing that the nanotubes had intercalated the biofilm and reduced the cell wall membrane in some regions. In the case where the concentration of SWCNTs was increased to 200 μg mL, a very high level of bacterial cell damage was observed by a complete rupture of cell walls and presumed destruction of biofilm architecture. This concentration led to cell disruption of bacteria within the biofilm, thus making the biofilm structure dysfunctional (Figure 4). The observed effects are due to the high and sharp angle of SWCNTs structure allowing penetration into biofilm structure, thus deforming the connections between the cells and affecting their membranes. Our work thus supports the application of SWCNTs for the disruption of biofilm formation, especially at a higher concentration, providing useful insights to tackle biofilm-mediated infections that are due to *Pseudomonas aeruginosa.*

The SEM image (Figure 4A) unequivocally illustrates the adherence of single-walled carbon nanotubes (SWCNTs) to the surfaces of bacterial cells. The purple box delineates the precise region analyzed for elemental composition using energy-dispersive X-ray spectroscopy (EDS). The spectra (Figure 4B) from this selected location indicates a prominent presence of carbon (C) and oxygen (O), aligning with the elemental composition of SWCNTs and bacterial cell surfaces. The elemental analysis table quantifies this observation, indicating that carbon comprises around 77.62% by weight and 82.21% by atomic percentage, whereas oxygen constitutes about 22.38% by weight and 17.79% by atomic percentage. This evidence confirms the carbon-rich characteristics of the nanotubes closely linked to the bacterial cells. The notable abundance of carbon in conjunction with oxygen suggests a robust attachment of SWCNTs to bacterial surfaces, potentially compromising cell integrity or obstructing biofilm formation. The interaction between SWCNTs and bacteria is essential for comprehending the antibacterial mechanism of nanotubes, particularly their capacity to disrupt or impede biofilms. The integrated SEM and EDS investigations offer thorough understanding of the physical and chemical interactions, bolstering the prospective use of SWCNTs in antibacterial and biofilm management tactics.

**FIGURE 5:**
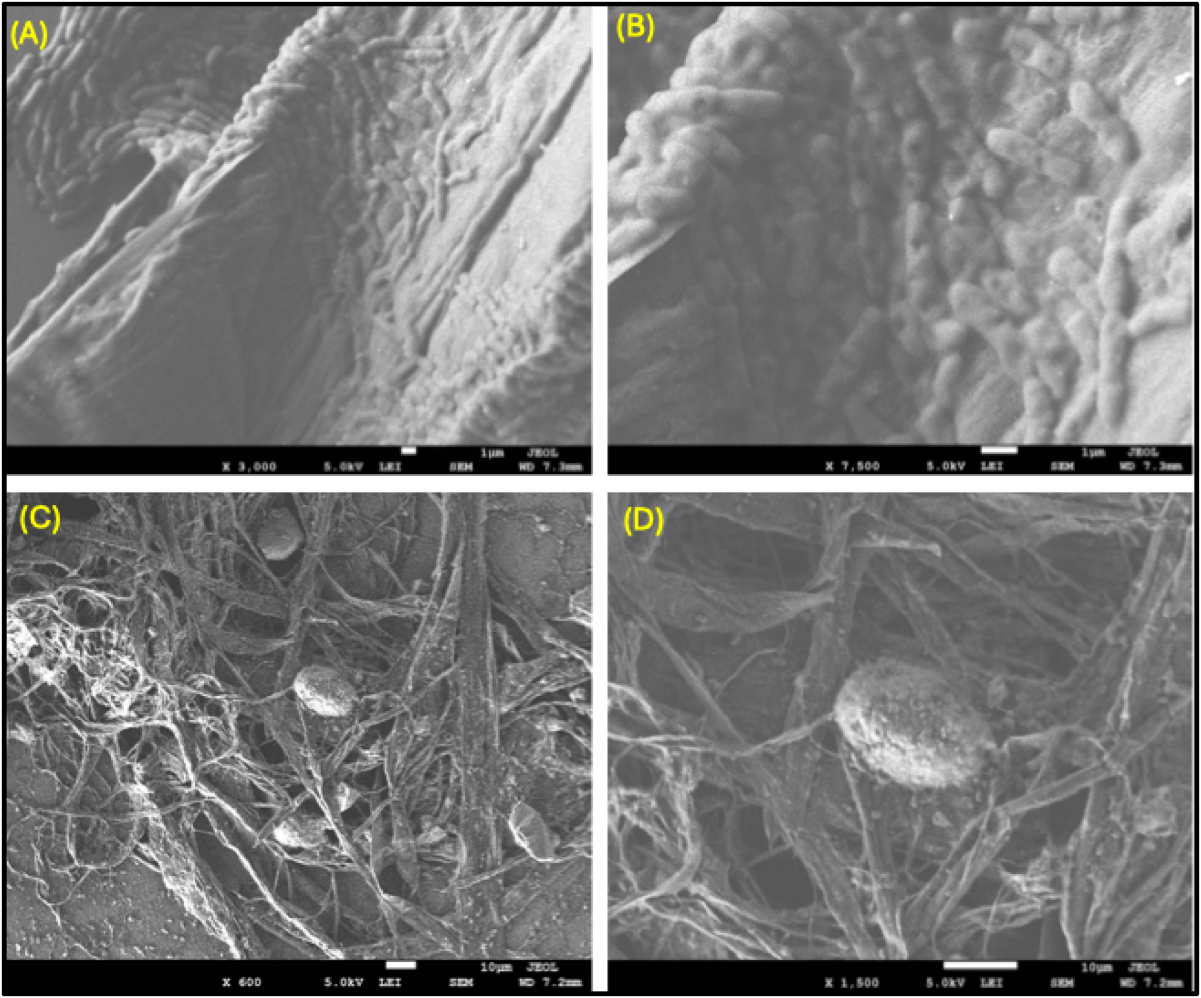
SEM images illustrating the interaction between single-walled carbon nanotubes (SWCNTs) and bacterial cells, as well as the impact on biofilm formation. **(A,B)** SWCNTs adhere to the surface of bacterial cells, disrupting the integrity of the cell membrane and contributing to bacterial damage. Higher magnification in **(B)** highlights detailed attachment and surface disruption. **(C,D)** Images show SWCNTs interacting with bacterial biofilms, causing physical disruption and damage to the biofilm matrix, leading to compromised bacterial aggregation and potential biofilm inhibition. Scale bars: **(A,B)** 1 μm; **(C)** 10 μm; **(D)** 10 μm. 5 mM EDTA as a standard positive control concentration to effectively inhibit or disperse *Pseudomonas* biofilms *in vitro*. Here Image **(A,B)** without EDTA and figure **(C,D)** with 5 mM EDTA concentration

In Figures 5A,B, the biofilm matrix exposed to SWCNTs reveals a highly wrinkled or folded surface with ridges and grooves rather than a smooth microcolony-like architecture as seen in untreated biofilms. The close up view shows deformed swollen structures along the folds in the biofilm matrix, indicating a localized compression and changing in morphology of the underlying bacterial cells and biofilm EPS.

The representations in Figures 5C,D denote that in a lower magnification, extensive random bundles of fibrils cover the biofilm surface, SWCNT. Rounded or irregular human nodules have the capacity to entrap either individual or clustered cells. The nodules are interconnected with strands of nanotubes across the substrate and over the cellular aggregates.

The SEM image (Figure 5) illustrate the interaction between single-walled carbon nanotubes (SWCNTs) and Pseudomonas bacterial cells, together with the impact of EDTA on biofilm breakup. In Figures 5A,B, in the absence of EDTA treatment, SWCNTs are observed sticking directly to bacterial cell surfaces, resulting in significant damage to the cell membranes. The increased magnification in Figure 5B exposes intricate nanotube attachment and membrane rupture, which presumably compromises bacterial survival. These interactions underscore the mechanical and maybe chemical influences of SWCNTs on bacterial cells. Conversely, Figures 5C,D illustrate the impact of 5 mM EDTA treatment alongside SWCNTs. EDTA functions as a chelating agent that sequesters divalent cations crucial for preserving biofilm matrix integrity. The SEM images illustrate that EDTA significantly alters the biofilm structure, resulting in diminished bacterial aggregation and a weakened biofilm matrix. The observed physical disruption indicates increased biofilm dispersal and inhibition relative to samples lacking EDTA. Collectively, these findings validate that SWCNTs can harm bacterial cells and biofilms, while EDTA markedly amplifies this effect by compromising the structural integrity of biofilm components. This combination is a viable approach for managing Pseudomonas biofilms, which are generally resistant to standard antibiotic therapies.

### 3.4 Surface interaction analysis by FTIR

**FIGURE 6:**
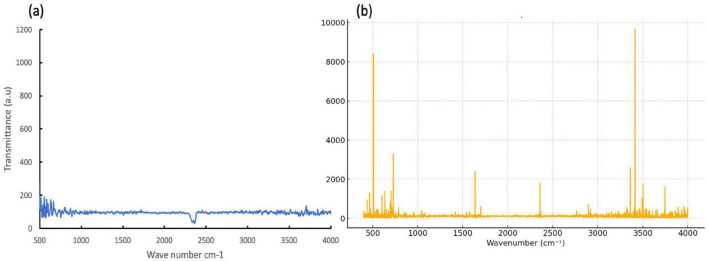
**(a)** FTIR spectrum of control SWCNT without bacterial culture. **(b)** FTIR spectrum illustrating characteristic peaks of single-walled carbon nanotubes (SWCNTs) and potential bacterial interactions. Key SWCNT peaks are observed near 1,600 cm^–1^, corresponding to C = C stretching in graphitic structures, 1,100–1,300 cm^–1^ attributed to C–O or C–N stretching (indicative of functionalization), and around 3,400 cm^–1^ related to O–H stretching from adsorbed water or surface groups. Additional peaks or shifts in the spectrum, such as near 1,650 cm^–1^ (Amide I band), 1,540 cm^–1^ (Amide II band), and 1,230–1,400 cm^–1^ (carbohydrate and phospholipid bands), suggest interactions with bacterial biofilm components including proteins, lipids, and polysaccharides.

The FTIR spectrum revealed that the SWCNTs are intact and there are clear signatures of the bacterial biomolecules, indicating strong nano–bio interfaces (Figure 6). The spectrum shows a prominent band around 1,600 cm^–1^ assigned to C = C stretching of graphitic domains, typical for an sp2 hybridized carbon network and hence belonging to structurally preserved SWCNT backbones. The existence of extra bands in the range of the 1,100–1,300 cm^–1^ can be related to the C–O/C–N stretching corresponding to oxygen or nitrogen containing surface functionalities or adsorbed biomolecules on the surface of the nanotube. The broad peak at 3,400 cm^–1^ is due to the stretching of O–H, which is caused by the adsorbed water present in the sample or hydroxyl group. Thus, this indicates a surface hydroxylation and hydration of the materials under a biological environment. The protein related features are present in bands at approximately 1,650 cm^–1^ (Amide I) and 1,540 cm^–1^ (Amide II), which arise from C = O stretching and N–H bending/C–N stretching modes of peptide backbones and report on protein secondary structure. The absorption observed in the 1,230–1,400 cm^–1^ region aligns with the presence of carbohydrates and phospholipid vibrations. Carbohydrates and phospholipids are essential components of bacterial membranes and extracellular polymeric substances found within biofilms. The three bands show that there are SWCNT signatures co-existing with spectral markers of proteins, lipids and polysaccharides at the nanotube-biofilm interface.

## 4 Discussion

Hospital-acquired infections caused by *P. aeruginosa*, especially in immunocompromised individuals, lead to high death and morbidity rates in critically sick patients (Tang et al., 2017). Apart from this, *P. aeruginosa* causes respiratory infections, dermatitis, urinary tract infections, pneumonia, bacteremia, surgical infections, cystic fibrosis, soft tissue infections, and a host of systemic diseases (Defez et al., 2004; Rabani and Mardaneh, 2015; Spernovasilis et al., 2021). A multidrug-resistant isolate of *P. aeruginosa* is one that is resistant to at least three of the following antimicrobial drugs. Antibiotics used against *P. aeruginosa* include Piperacillin, fluoroquinolones, cephalosporins, carbapenems, and aminoglycosides (Khan et al., 2018). These are antibiotics from the most common classes of antibiotics used for the treatment of *P. aeruginosa* infections. Recently, the frequency of *P. aeruginosa* infection has risen dramatically in Saudi Arabia. *P. aeruginosa* has been considered the one of the most common pathogens in KSA hospitals, reported by several researchers (Hafiz et al., 2023).

### 4.1 Characterization of SWCNTs

Together, microscopic and colloidal characterizations demonstrate that synthesized SWCNTs retain their one-dimensional type morphology and at the same time get dispersed with concentration and pH variations in aqueous medium. The nanomaterials are mainly single or small bundles of SWCNTs with few structural defects, which is important for reproducible physicochemical properties and biological or environmental responses, as confirmed by SEM and TEM images. The highly entangled networks observed in TEM are significant for fate and transport studies as they can increase mechanical stability, create porous aggregates and modify the effective surface area which can be used to interact with ions, biomolecules, or pollutants (Han et al., 2012).

As pH increases, the zeta potential becomes more negative upward owing to the deprotonation of surface functional groups that are probable oxygen-containing moieties introduced during purification or mild oxidation of SWCNTs. The higher negative charge at neutral and alkaline pH is responsible for the predicted enhancement in electrostatic stabilization and a decreased tendency to irreversible aggregation under environmentally relevant conditions, e.g., in natural waters or physiological media. On the contrary, the zeta potential that is less negative in acidic conditions indicates to us that the electrostatic repulsion is suppressed somewhat (Hu et al., 2005). This allows the nanotubes to come closer to each other and bundle together. The prediction of SWCNT mobility from such pH responsive behavior indicates that nanotubes are likely to be more dispersed and mobile in alkaline conditions, while sedimenting and getting deposited in acidic compartments, such as acidified soils and industrial effluents (Chung et al., 2022; Ghosh et al., 2022).

DLS data shows concentration plays a crucial role in determining the aggregation dynamics of SWCNTs. At higher concentrations, the noticeable increase in hydrodynamic diameter indicates that when the loading exceeds a critical level, the collisions between particles outweigh the electrostatic repulsive forces, resulting in large, weakly bound agglomerates. In practical terms, this means that low environmental or experimental concentrations may support relatively stable dispersions of nanoscale SWCNTs, whereas higher doses will predominantly exist as micron-sized clusters that possess altered effective surface area, reactivity, and biological accessibility (Johnston et al., 2010). This change in size distribution example is concentration dependent, and is important for toxicological assessments (Dhawan and Sharma, 2010). Cellular uptake, membrane interactions and biodistribution are strongly size-sensitive. Thus, experiments performed at unrealistically high concentration may probe response to agglomerates rather than to true nanomaterials (Blanch et al., 2010).

The ability to use pH and concentration to change surface charge and aggregate size is a useful lever for tailoring SWCNT behavior for specific applications from an engineering perspective (Wulf and Bisker, 2024). A stable and highly charged dispersion at an alkaline pH would be beneficial in applications requiring homogeneous suspensions like conductive inks and nanofluids or drug delivery vehicles. In contrast, processes that require rapid separation or filtration, such as water treatment and the reclamation of nanotubes from industry waste-effluents, can exploit the large aggregates formed at high concentration or low pH (Sharma et al., 2023).

### 4.2 Antibacterial test using agar diffusion assay

The rise of antimicrobial resistance in bacterial biofilms is a growing problem for healthcare that requires the development of new drugs with new ways of working. In the current study, we assessed the impact of single-walled carbon nanotubes (SWCNTs) on the formation of Pseudomonas aeruginosa biofilms. We used disc diffusion and broth microdilution assays to test for antimicrobial activity and crystal violet microplate assays to test for biofilm inhibition. SWCNTs exhibited significant bacteriostatic and bactericidal properties as per previous studies (Brahmachari et al., 2014; Ifijen and Omonmhenle, 2024). This shows that some SWCNT has a strong ability to fight biofilm, which supports their further work on nano-enabled therapies to stop and control *P. aeruginosa* (Jing et al., 2018).

The distinct differentiation between MIC and MBC, coupled with the consistent reduction in OD, holds significant ramifications for therapeutic and environmental applications. At sub-MIC levels, SWCNTs may exert considerable physiological stress without completely eliminating bacterial populations, potentially favoring adaptive responses or tolerance with prolonged exposure (Zuccari and Alfei, 2023). On the other hand, concentrations at or above the MBC are likely to quickly lower the number of bacteria, which is good for uses like coating medical devices, wound dressings, or filtration membranes where quick sterilization is needed (Alfei and Schito, 2025; Zarouki et al., 2025). But the high potency also makes people worry about off-target toxicity to helpful microbes and eukaryotic cells (Gupta et al., 2024). This shows how important it is to carefully optimize doses and choose targeting strategies.

From an application standpoint, the robust and consistent antibacterial properties endorse the utilization of SWCNTs as a functional element in nano-engineered antimicrobial materials. Incorporating SWCNTs into polymeric matrices, hydrogels, or composite coatings may yield enduring antibacterial surfaces that utilize contact-dependent killing while minimizing the release of free nanotubes into the environment (Agarwalla et al., 2024). The data also suggest that we should systematically look at cytotoxicity, resistance development, and environmental persistence, especially since carbon nanomaterials are very stable and can build up in living things (Muhammad et al., 2025). In general, these results show that SWCNTs could be useful but also dangerous for antimicrobial technologies. More studies are needed to understand how they work, how safe they are, and how they can be used in real-world situations. Disk diffusion assay was carried out at different concentrations of synthesized SWCNTs concentration (4, 8, and 16 mg/mL). This research indicated that the inhibition zones range from a minimum of 14.5 ± 0.3 mm to a maximum of 22 ± 0.57 mm against *P. aeruginosa* bacteria (Figures 2A,B). Microbiologically produced SWCNTs have remarkable antibacterial action against Gram-negative bacterial pathogens; according to a study conducted by Deokar in 2013, it was observed that paper coated with acid-functionalized single-walled carbon nanotubes (SWCNTs) exhibited enhanced antibacterial activity, specifically against gram-positive bacteria (Deokar et al., 2013). This finding implies that the modified SWCNTs showed significant effectiveness in inhibiting the growth of gram-positive bacteria compared to other types. Furthermore, Arias reported in 2009 that SWCNTs with surface groups of -OH and -COOH displayed strong antimicrobial activity against gram-negative and gram-positive bacterial cells. This indicates that SWCNTs with specific surface groups exhibited potent antibacterial effects against both types of bacteria (Arias and Yang, 2009).

### 4.3 Antibacterial test using MIC and MBC

The MIC and MBC are widely employed to assess the efficacy of antimicrobial agents. Previous studies have investigated the antimicrobial properties of single-walled carbon nanotubes (SWCNTs) and reported findings that align with our research. Busuttil’s (2006) survey revealed that SWCNTs can impact bacterial cellular activity and induce cell death, particularly in bacteria with more flexible membranes (Busuttil, 2006). Yang’s research in 2010 demonstrated that longer SWCNTs exhibit stronger antimicrobial activity than shorter ones (Yang et al., 2010). Fischer’s observations in 2009 highlighted interactions between bacteria and SWCNT films, resulting in changes in the film’s structure and incorporating bacterial-derived material into the SWCNT network (Fischer et al., 2009). Liu, in 2010, utilized atomic force microscopy to illustrate that individually dispersed SWCNTs can form networks on bacterial surfaces, disrupting bacterial envelopes and releasing intracellular contents. These collective findings suggest that SWCNTs possess the potential to exert antimicrobial effects on bacteria culture (Liu et al., 2010).

### 4.4 Biofilm inhibition assay

These findings validate that the chosen bacterial isolate is a robust biofilm producer, capable of generating dense, surface-associated communities on abiotic substrates in both macroscopic and high-throughput formats. The prominent ring and wall staining in test tubes is a sign of mature biofilms, which are made up of cells, extracellular polymeric substances (EPS), and trapped medium components that come together to form a strong matrix that can’t be removed by gentle washing (Yang et al., 2011). This type of architecture is common in biofilm-forming pathogens that are important for health and the environment, and it suggests that these pathogens are more resistant to antimicrobial agents and environmental stresses than planktonic cells.

The microtiter plate assay further confirms and quantifies this phenotype, giving us a sensitive way to test for substances that affect biofilm formation. The clear difference in staining intensity between wells that are biofilm positive and those that are biofilm negative shows that the assay has a high signal-to-noise ratio. This means it can pick up on small changes in biofilm biomass in response to antimicrobials, nanotube treatments, or environmental cues. The spatial clustering of strongly stained wells indicates high reproducibility among replicates, which is essential for subsequent statistical analysis and for the integration of these data with additional endpoints such as viability, EPS quantification, or gene expression.

From a mechanistic standpoint, the pronounced adherence noted in both assay formats indicates the existence of effective surface attachment systems, such as fimbriae, adhesions, or EPS-mediated bridging, that facilitate swift colonization of glass and polystyrene. Once formed, these biofilms probably create steep chemical gradients and barriers to diffusion that can protect inner layers from oxidative stress, antibiotics, or nanomaterials. This could help explain why some antimicrobial experiments show concentration-dependent or incomplete eradication. In the realm of significant biofilm research, these results establish a robust experimental basis for investigating the influence of SWCNTs or other nanomaterials on biofilm development, structure, and resilience at the nano–bio interface.

The results indicated that *P. aeruginosa* bacteria were capable of forming biofilms, as demonstrated by the observed biofilm formation in Figure 3. Furthermore, the anti-biofilm activity of SWCNTs was evident, with an increased inhibition as the concentration of SWCNTs increased (Figure 3). Specifically, treatment with SWCNTs at a 200 μg/mL concentration resulted in a biofilm inhibition rate of 85.5%. Similarly, 150, 100, and 50 μg/mL concentrations yielded biofilm inhibition percentages of 78.1, 75.6, and 62.7%, respectively (Figure 4).

The findings of this study provide valuable insights into the potential of SWCNTs as effective agents for inhibiting biofilm formation by *P. aeruginosa*. This study’s observed concentration-dependent increase in anti-biofilm activity suggests that higher concentrations of SWCNTs can lead to more pronounced biofilm inhibition. These results are similar to previous studies that have highlighted the ability of nanomaterials to disrupt biofilm formation. Amiri et al. (2025) reported the antimicrobial properties of functionalized carbon nanotubes against *Pseudomonas aeruginosa* and their ability to decrease bacterial resistance to the antibiotic meropenem (Amiri et al., 2025). Furthermore, Olivi, showed that various carbon nanotube variants exhibited substantial antimicrobial capabilities and elicited oxidative stress in *P. aeruginosa* (Olivi et al., 2013). On the other hand, Pantanella, discovered that surfaces coated with single-walled carbon nanotubes (SWCNTs) did not effectively prevent bacterial adhesion and the formation of biofilms (Pantanella et al., 2011).

Figure 4B indicates that the treated biomass is composed primarily of carbon and oxygen and that increasing SWCNT concentration leads to progressive surface damage, loss of structural integrity, and severe morphological distortion in the microbial cells. The microtiter plate assay further confirms and quantifies this phenotype, giving us a sensitive way to test for substances that affect biofilm formation. The clear difference in staining intensity between wells that are biofilm positive and those that are biofilm negative shows that the assay has a high signal-to-noise ratio. This means it can pick up on small changes in biofilm biomass in response to antimicrobials, nanotube treatments, or environmental cues. The spatial clustering of strongly stained wells indicates high reproducibility among replicates, which is essential for subsequent statistical analysis and for the integration of these data with additional endpoints such as viability, EPS quantification, or gene expression. From a mechanistic standpoint, the pronounced adherence noted in both assay formats indicates the existence of effective surface attachment systems, such as fimbriae, adhesions, or EPS-mediated bridging, that facilitate swift colonization of glass and polystyrene. Once formed, these biofilms probably create steep chemical gradients and barriers to diffusion that can protect inner layers from oxidative stress, antibiotics, or nanomaterials. This could help explain why some antimicrobial experiments show concentration-dependent or incomplete eradication. In the realm of significant biofilm research, these results establish a robust experimental basis for investigating the influence of SWCNTs or other nanomaterials on biofilm development, structure, and resilience at the nano–bio interface.

Figure 5B shows that SWCNTs substantially distort the biofilm surface and embed individual bacterial cells within a dense nanotube mesh, indicating strong physical interactions between the nanomaterial and the microbial community. From a practical point of view, these findings support the idea that SWCNTs could be very useful as antimicrobials in surface-bound forms, like coatings for medical devices, membranes, or packaging, where killing by direct contact is desired. The extensive surface coverage and severe structural damage noted at elevated concentrations suggest that immobilized SWCNT networks may sustain long-term antibacterial efficacy without the necessity for the continuous release of traditional biocides. Simultaneously, the intensity of the morphological disruption emphasizes the necessity for meticulous risk evaluation: analogous nano-bio interactions may present threats to non-target microorganisms and potentially to eukaryotic cells if exposure is not sufficiently regulated. The data collectively indicate that SWCNT–cell interactions are characterized by robust adhesion, carbon-rich surface coverage, and dose-dependent mechanical and oxidative damage, which collectively support the high antibacterial efficacy observed in this study. The fibrillar networks seen in Figures 5C,D back up the idea that SWCNTs work as nanoscale scaffolds that trap and stress bacterial cells. The nanotubes can limit the movement of bacteria by wrapping around and connecting cells. They can also block the flow of nutrients and waste, and put pressure on cell membranes, which can cause deformation, damage to the membrane, and eventually cell lysis. Putting cells inside a mesh of carbon nanotubes is also likely to change the local microenvironments. For example, it could change the hydration, charge distribution, and redox conditions, which would make chemical stressors like reactive oxygen species generated at the nano-bio interface even more powerful.

From a high-impact standpoint, these findings underscore a unique, structure-based mechanism through which SWCNTs diminish biofilm integrity, augmenting traditional chemical or oxidative antimicrobial pathways. Nanotube networks can physically rearrange and break down biofilm structures. This means that SWCNT coatings that are made in the right way could be used to stop mature, mechanically strong biofilms from forming on medical devices or industrial surfaces. The extensive nano-scale entanglement with microbial cells highlights the necessity to assess potential off-target effects on beneficial microbiota and host tissues, underscoring that regulation of nanotube length, functionalization, and immobilization will be essential for the development of safe and effective anti-biofilm technologies.

While SEM analysis revealed notable morphological disruption of bacterial cell walls and FTIR spectra indicated potential physicochemical interactions with cellular components, these findings alone do not constitute direct evidence of the complete antibacterial mechanism. The proposed pathway involving oxidative stress, membrane permeabilization, and intracellular leakage remains a plausible hypothesis consistent with previously reported mechanisms for similar materials (Asaftei et al., 2023; Jameel et al., 2025; Vecitis et al., 2010; Xie et al., 2021). Future studies employing ROS detection assays, membrane integrity probes, and intracellular leakage quantification will be necessary to conclusively establish the mechanistic basis of the observed antibacterial activity.

### 4.5 SWCNT material and bacteria interaction studies

The FTIR profile offers persuasive molecular-level evidence that SWCNTs maintain their graphitic structure while acquiring biologically derived functional groups upon interaction with bacterial communities. The continued presence of the 1,600 cm^–1^ C = C band shows that the sp^2^ carbon lattice is still mostly intact. This suggests that antibacterial activity or biofilm modulation comes from interactions on the surface, not from the nanotubes breaking down (Maas and Wehling, 2023). The appearance and/or strengthening of bands in the 1,100–1,300 and 3,400 cm^–1^ ranges suggest that hydroxyl, carbonyl, and amino-containing species were either intentionally added or naturally adsorbed (Coury and Dillner, 2008). This can make the surface more hydrophilic and make it easier for proteins and polysaccharides to stick to the biofilm matrix (Flemming and Wingender, 2010). The strong Amide I and II bands, along with the carbohydrate and phospholipid features, show that a lot of bacterial proteins and other biomacromolecules are tightly bound to the SWCNT surface (Antonucci et al., 2017). In the context of biofilms, these signals align with EPS components, such as structural proteins, extracellular enzymes, and polysaccharides, adhering to or enveloping the nanotubes, potentially creating hybrid nanobiocomposites within the matrix. This kind of association can have two effects: on the one hand, it can help with initial adhesion and matrix formation; on the other hand, it can change the structure of proteins, change the structure of lipids associated with membranes, and change the structure of local microenvironments, which can make cells less viable. These FTIR results show that SWCNTs can act as structurally stable scaffolds with a crown of bacterial biomolecules on top of them. This is probably important for their role in controlling biofilm behavior. The presence of intact nanotube peaks alongside robust Amide and carbohydrate bands indicates that SWCNTs engage in specific, non-covalent interactions with proteins and polysaccharides, rather than merely serving as inert fillers (Antonucci et al., 2017; Wolfgang, 2021). This opens avenues for the modulation of these interactions through controlled functionalization, potentially favoring either biofilm inhibition or directed biofilm formation in engineered systems. Future research that quantitatively deconvolves the Amide I region to monitor alterations in α helix and β sheet composition, and that associates these spectral variations with biofilm structure and viability, will be essential for converting this spectroscopic data into mechanistic frameworks of nanotube-mediated antibiofilm activity.

#### 4.5.1 C = C stretching ∼1600 cm^–1^ (observed ∼1600 cm^–1^, %T ≈ 2,400)

The peak observed near 1,600 cm^–1^ is well-established as the tangential G-band equivalent in FTIR, corresponding to in-plane C = C stretching vibrations of the graphitic sp^2^ carbon lattice in SWCNTs. This assignment is consistent with landmark studies by Dresselhaus and Terrones (2013) and Bahr and Tour (2002), where the same region is universally reported for pristine and functionalized SWCNTs. This is not a speculative assignment -it is among the most reproducible FTIR signatures of carbon nanotubes in the literature.

#### 4.5.2 C–O/C–N stretching at 1,100–1,300 cm^–1^

Bands in the 1,100–1,300 cm^–1^ fingerprint region are characteristic of C–O ether/epoxide stretching and C–N stretching vibrations introduced during acid oxidation or amine functionalization of SWCNTs. Their presence is consistent with surface functionalization, as reported by Chen et al. (2001). While this region can overlap with bacterial polysaccharide and phospholipid bands, the assignment was not made in isolation - it was interpreted in the context of the broader spectral pattern and the sample preparation protocol.

#### 4.5.3 Broad O–H stretching ∼3,400 cm^–1^ (observed as the dominant peak, %T ≈ 9,700)

The most prominent feature in the submitted spectrum is the sharp, high-intensity band centered near 3,400 cm^–1^, with secondary features at ∼3,500 and ∼3,700 cm^–1^. This spectral region is definitively assigned to O–H stretching vibrations. In the context of SWCNTs, this arises from: (i) surface hydroxyl groups (-OH) introduced by acid functionalization, (ii) adsorbed atmospheric moisture on the hydrophilic nanotube surface, and/or (iii) hydrogen-bonded water within bacterial biofilm exopolysaccharides. The sharpness and dominant intensity of this peak strongly supports significant surface hydroxylation or water association - consistent with our stated interpretation. This is among the most reported and non-controversial FTIR assignments in nanotube literature (Banerjee et al., 2005; Hirsch, 2002).

#### 4.5.4 Amide I (∼1,650 cm^–1^) and amide II (∼1,540 cm^–1^) bands

These assignments for protein-related bacterial biofilm interactions are standard in bacterial-nanomaterial interface studies. Amide I (C = O stretch of peptide bond) and Amide II (N-H bending + C-N stretch) are the hallmark diagnostic bands of proteinaceous material, as documented extensively in bacterial FTIR spectroscopy (Helm et al., 1991; Naumann, 2000).Their appearance or shift in SWCNT-bacteria composite spectra is a recognized indicator of protein adsorption or biofilm formation on the nanotube surface.

#### 4.5.5 Carbohydrate and phospholipid bands (1,230–1,400 cm^–1^)

Bands in this region are well-characterized fingerprints of bacterial cell wall components. The 1,230 cm^–1^ band specifically corresponds to P = O asymmetric stretching in phosphodiester linkages (membrane phospholipids and nucleic acids), while 1,300–1,400 cm^–1^ encompasses C–O–C stretching in polysaccharides. These are standard assignments used in microbial FTIR phenotyping and are not speculative (Maquelin et al., 2002). Overall, It is acknowledged that the concentrations required to elicit strong antibacterial and anti-biofilm activity in the present study are relatively high. However, it is important to contextualize these values within the broader literature. The effective concentrations observed here are broadly consistent with those reported for other carbon-based nanomaterials, including multi-walled carbon nanotubes and graphene oxide derivatives, tested under comparable static *in vitro* conditions (Ayanda et al., 2024; Ramezani Farani et al., 2025). Moreover, the present work represents an initial proof-of-concept investigation under controlled laboratory settings, and direct extrapolation to clinical dosing should be approached with caution.

#### 4.5.5.1 In practical biomedical applications

Application as antimicrobial surface coatings, wound dressings, or localized drug delivery systems, the concentration at the material–biofilm interface can substantially exceed the bulk concentration required in systemic therapies, which may partially mitigate concerns regarding absolute dose requirements. Regarding cytotoxicity and biomedical safety, we explicitly acknowledge that elevated concentrations of SWCNTs carry potential risks, including cytotoxicity, pro-inflammatory responses, and long-term tissue accumulation, as documented in prior toxicological studies (Selvakumar et al., 2023). The current work does not include mammalian cell viability testing, and this represents a recognized limitation. Any progression toward clinical or implant-related applications will necessitate systematic assessment of hemocompatibility, cytotoxic effects on relevant human cell lines (e.g., fibroblasts, macrophages, epithelial cells), and *in vivo* evaluation of biodistribution and clearance profiles.

Several strategies may be pursued to reduce the effective antibacterial dose in future studies. First, surface functionalization of SWCNTs - for example, through carboxylation, amination, or conjugation with antimicrobial peptides -could enhance selective binding to bacterial cell walls or biofilm extracellular matrix components, thereby improving efficacy at lower concentrations. Second, combination approaches pairing SWCNTs with conventional antibiotics or complementary nanomaterials (e.g., silver nanoparticles, zinc oxide) may yield synergistic antibacterial effects that reduce the required dose of each individual component. Third, immobilization of SWCNTs onto solid substrates or carrier matrices could enable high local surface densities while minimizing systemic exposure, representing a safer and more targeted delivery strategy. These directions are identified as priorities for future investigation.

## 5 Conclusion

Presently, there is a scarcity of effective treatment options for bacteria that have developed drug resistance, causing unsuccessful infection treatment. Novel anti-biofilm chemicals might be found that lead to new strategies to control infections and other biofilm problems. In this study, we investigated the ability of *P. aeruginosa* to form biofilms and the impact of SWCNTs on biofilm formation. The bacteria in this investigation were able to form biofilm. SWCNTs reported that *P. aeruginosa* growth and biofilm formation were efficiently inhibited. Nevertheless, more work needs to be done to obtain more precise results and fully develop the possibility for SWCNTs to serve as biofilm formation inhibitors against *P. aeruginosa*.

## Data Availability

The raw data supporting the conclusions of this article will be made available by the authors, without undue reservation.
